# Use of Complementary and Alternative Medicine (CAM) in cancer patients: An Italian multicenter survey

**DOI:** 10.18632/oncotarget.14224

**Published:** 2016-12-25

**Authors:** Massimiliano Berretta, Chiara Della Pepa, Paolo Tralongo, Alberto Fulvi, Ferdinando Martellotta, Arben Lleshi, Guglielmo Nasti, Rossella Fisichella, Carmela Romano, Chiara De Divitiis, Rosaria Taibi, Francesco Fiorica, Raffaele Di Francia, Anna Di Mari, Lino Del Pup, Anna Crispo, Paolo De Paoli, Adriano Santorelli, Vincenzo Quagliariello, Rosario Vincenzo Iaffaioli, Umberto Tirelli, Gaetano Facchini

**Affiliations:** ^1^ Department of Medical Oncology, National Cancer Institute, Aviano (PN), Italy; ^2^ Division of Medical Oncology, Department of Uro-Gynaecological Oncology, Istituto Nazionale Tumori IRCCS “Fondazione G. Pascale”, Naples, Italy; ^3^ Division of Medical Oncology, “Umberto I” Hospital, Syracuse, Italy; ^4^ Department of Medicine and Surgery of Integrated Treatment, Division of Thoracic Oncology, “San Camillo Forlanini” Hospital, Rome, Italy; ^5^ Department of Abdominal Oncology, Division of Medical Oncology B, National Cancer Institute, “G. Pascale” Foundation, Naples, Italy; ^6^ Department of Surgery, University of Catania, Catania, Italy; ^7^ Department of Radiation Oncology University Hospital Ferrara, Division of Radiotherapy, “Arcispedale Sant’Anna” Hospital, Ferrara, Italy; ^8^ Department of Hematology, Istituto Nazionale Tumori ‘Fondazione Giovanni Pascale’, IRCCS, Naples, Italy; ^9^ Gruppo Oncologico Ricercatori Italiani, GORI, Pordenone, Italy; ^10^ Division of Gynaecological Oncology, National Cancer Institute, Aviano (PN), Italy; ^11^ Unit of Epidemiology, Struttura Complessa di Statistica Medica, Biometria e Bioinformatica, Istituto Nazionale Tumori IRCCS “Fondazione G. Pascale”, Naples, Italy; ^12^ Scientific Directorate, National Cancer Institute, Aviano (PN), Italy; ^13^ Department of Plastic Surgery, Regenerative Medicine, Health Park Hospital, Naples, Italy

**Keywords:** complementary medicine, alternative medicine, survey, cancer, treatment

## Abstract

**Introduction:**

Complementary and Alternative Medicine (CAM) include a wide range of products (herbs, vitamins, minerals, and probiotics) and medical practices, developed outside of the mainstream Western medicine. Patients with cancer are more likely to resort to CAM first or then in their disease history; the potential side effects as well as the costs of such practices are largely underestimated.

**Patients and method:**

We conducted a descriptive survey in five Italian hospitals involving 468 patients with different malignancies. The survey consisted of a forty-two question questionnaire, patients were eligible if they were Italian-speaking and receiving an anticancer treatment at the time of the survey or had received an anticancer treatment no more than three years before participating in the survey.

**RESULTS:**

Of our patients, 48.9% said they use or have recently used CAM. The univariate analysis showed that female gender, high education, receiving treatment in a highly specialized institute and receiving chemotherapy are associated with CAM use; at the multivariate analysis high education (Odds Ratio, (OR): 1.96 95% Confidence Interval, CI, 1.27-3.05) and receiving treatment in a specialized cancer center (OR: 2.75 95% CI, 1.53-4.94) were confirmed as risk factors for CAM use.

**Conclusion:**

Roughly half of our patients receiving treatment for cancer use CAM. It is necessary that health professional explore the use of CAM with their cancer patients, educate them about potentially beneficial therapies in light of the limited available evidence of effectiveness, and work towards an integrated model of health-care provision.

## INTRODUCTION

According to the National Center for Complementary and Integrative Health, USA, the Complementary and Alternative Medicine (CAM), include a wide range of products, such as herbs (also known as botanicals), vitamins, minerals, and probiotics, and medical practices, such as acupuncture or magneto-therapy, which are outside of the mainstream Western medicine. Such practices or substances are defined ‘alternative’ when they are used *in place of* conventional medicine and ‘complementary’ when they are used *together with* conventional medicine [1]. The interpretation provided by Ernst E et al. (UK), is much more complex and defines CAM as ‘diagnosis, treatment and/or prevention which complements mainstream medicine by contributing to a common whole, satisfying a demand not met by orthodoxy, or diversifying the conceptual framework of medicine’ [2]. Both the definitions are correct and one does not exclude the other; indeed the use of such unconventional substances/treatments, which has always been very common among the eastern populations and in the developing countries, is acquiring more and more popularity also in the western world for multiple reasons. In the US, the use of CAM is constantly increasing with the largest practice being among the non-hispanic whites [3] and in the rural areas (rate of use up to 63%) compared with the cities [4, 5]; in terms of geographic areas the highest rate of use was observed in the Mountain Regions and in New Mexico [6]. A systematic review published in 2013 by Posadzki P et al. highlighted the difficulties of identifying reliable data about the prevalence of CAM due to the poor methodological quality of most studies and the ambiguity of the definition of CAM; however, the authors concluded that the average one-year prevalence is 41.1% and the average lifetime prevalence is 58.1% [7]. A European survey conducted by Molassiotis A. et al., demonstrated that the use of CAM in the EU is about 35.9% of the total patient population. The use of CAM has increased steadily over the past 15 years or so; undoubtedly it has gained medical, economic and social importance [8]. Patients with cancer are more likely to resort to CAM, first or then in their disease history, for a wide range of reasons; firstly, the unfavorable outcome in a relevant percentage of cases leads patients to ‘leave no stone unturned’; secondly, the heavy toxicities, often associated with the traditional antineoplastic therapies, induce them to look for something different from the prescribed therapy or more simply for substances presumed to reduce the side effects from such therapies [9]. Nevertheless the literature about CAM prevalence in cancer patients is not particularly rich, especially if we consider only the European papers, and the prevalence is probably underestimated. Many patients do not declare that they engage in this practice, on the one hand because they undervalue the relevance of the products they take, considering them ‘natural’, unable to interact with the conventional drugs and devoid of side effects, and, on the other hand, because they are somehow reluctant to admit the use of an unconventional treatment, worrying that such behavior may be interpreted as reflecting a loss of trust in their oncologist and the treatment he/she has prescribed. Furthermore most clinicians are unfamiliar with these kinds of treatments [10] and hence do not pay enough attention to this aspect of the anamnesis at the time of the visit; usually they do not explicitly ask about this topic, as they do for all other health matters such as comorbidity or conventional drugs [9, 11]. The available studies report that the prevalence of CAM use among cancer patients is in the range of 12.5-73% [12–15]. This enormous variability is, at least partially, justified by the inconsistent definition of CAM, with some authors including only herbal medications, while some others considering also including dietary supplements and unconventional medical practices (massages, acupuncture). The aim of this study was to assess the use of CAM across a number of Italian cancer hospitals, using the same measurement tool and the same definition of CAM and trace the “identikit” of typical cancer patient “CAM-users”.

## RESULTS

The five hospitals that participated in the study provided a total of 468 subjects that were eligible for clinical considerations and statistical analysis. Table 1 summarizes the baseline characteristics of the population.

**Table 1:** Baseline and demographics

Baseline and Demographics n=468	
***SEX***	***n (%)***
male	229(48.9)
female	239(51.1)
***Median Age at diagnosis (range)***	57 (19-82)
***Age***	
< 40	23 (4.9)
40 – 70	245 (52.4)
> 70	200 (42.7)
***EDUCATION***	
Elementary/middle (< 8 ys)	178 (38)
High/degree (> 8 ys)	290 (62)
***ORIGIN***	
Centre/North	245 (52.4)
South/Islands	223 (47.6)
***HOSPITAL***	
Cancer centre	325 (69.4)
Peripheral hospital/clinic	143 (30.6)
***PRIMARY TUMOUR***	
Lung	222 (47.4)
Breast	78 (16.7)
Colorectal	56 (12)
Gastric/Pancreas/HCC/Biliary tract	23 (4.9)
Uro-Gynecology	26 (5.6)
Other	63(13.5)
***STAGE***	
Early	100 (21.4)
Locally advanced	114 (24.4)
Metastatic	254 (54.3)
***TREATMENT***	
AC only	238 (50.9)
Multimodal	154 (32.9)
Surgery only	25 (5.3)
Surgery + AC	51 (10.9)
***ANXIETY***	
Never	314 (67.1)
Sometimes	82 (17.5)
Often	72 (15.4)
***PAIN***	
No	318 (67.9)
Yes	150 (32.1)
***PS***	
1-2	346 (73.9)
> 2	69 (14.7)
0	53 (11.3)

**Legend:** ys, years; AC, Chemotherapy; Multimodal: surgery +chemotherapy + hormone therapy/radiation or both; PS, Performance Status.

### Socio-demographic and clinical characteristics of the sample

With respect to demographics, the cohort was well balanced between males and females (48.9% vs. 51.1%), the median age at the time of diagnosis of cancer was 57 years old, while the age at the time of the survey was between 40 and 70 years of age for more than half of the patients. The educational level was low (primary or secondary school) for 38%, high (high school, degree or higher) for 62%; the Italian region of origin was the South or the Islands for 47.6%, and the Centre or North for the remaining 52.4%. Most patients received their treatment at a cancer center rather than at a peripheral hospital or clinic (69.4% vs 30.6%). With respect to disease characteristics, the primary cancer site was lung (47.4%), followed by breast (16.7%), and colorectal (12%). Most patients had a metastatic (54.3%), or locally-advanced (24.4%), disease; the treatment received was based on AC alone in the 50.9% of the cases, a good percentage, 32.9%, received a multimodal treatment (surgery plus AC plus hormone therapy and/or radiotherapy), only 25 (5.3%) had only surgery. Most subjects declared that they did not anxiety disorders, 67.1%, and did not complain of chronic pain, 67.9%. The ECOG Performance Status was 1 or 2 in most cases (73.9%).

### Information about CAM

**Figure 1:**
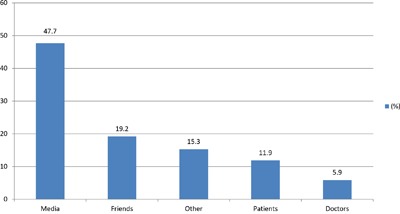
Source of knowledge about CAM

Table 2 shows the answers about the general feeling with respect to conventional medicine and CAM: More than 90% thought they had a good/excellent awareness about the disease and the therapies received. Almost all the interviewed declared that they have trust in conventional medicine and oncological treatments, 94.2% and 95% respectively, though roughly 50% expected to be treated rather than cured and there was a relevant percentage, 32%, admitting to be concerned about financial speculation around anticancer drugs. Of the population surveyed, 75.6% said they knew what CAM was, but only 27.6% were aware of the difference between alternative and complementary medicine. Among the patients aware of what CAM was (354), most had heard about it from the media, friends, or other patients, and only the 5.9% from a doctor (Figure 1). Eight patients (3.5%) reported side-effects from the CAM therapy assumption. Most seemed to be transient side-effects and they were all related to ingesting herbs or minerals. These side effects included stomach aches (two with aloe, one with vitamin C and two with high consumption of green tea) gastric upset and nausea from using unspecified herbs (two patients), and diarrhoea with aloe (one patient). The most frequent answer to the question ‘what CAM is?’ was ‘medicines to reduce AC toxicities’ followed by ‘supplements’ and ‘overall’, which meant ‘all the above’. Of the subjects, 48.9% (229) answered ‘yes’ to the direct question ‘have you ever used any CAM?’. Table 3 shows the distribution of CAM users by type, prescriber, cost, and satisfaction with the product. Interestingly, the use of CAM mainly derived from an auto-prescription (67%), most patients were not aware of the potential side effects, the substance was bought by the patient himself at an average cost of between 100 and 300 euros, the duration of the treatment was less than one year in 83.8% of the cases. Evaluating the variables associated with CAM use through the univariate analysis, we noticed that it was more popular among females compared with males (56.9 and 44.5%, p= 0.01), and older patients, ≥ 70 years of age, compared with younger, 40-70, (54.6 and 42.4%, p< 0.001). Also, a medium-high educational level seemed more frequently associated with CAM use compared to a low one (74.2 vs. 25.8%, p < 0.001). Looking at the primary disease, it appeared that patients with a more aggressive malignancy, in particular lung cancer, use CAM more frequently compared to the others; furthermore, CAM was resulted more popular among subjects receiving AC alone [139 patients (60.7%)] rather than other treatment modalities. Other characteristics associated with CAM use include anxiety disorders, lack of chronic pain, and metastatic disease (Table 4). Of note is that there was no difference in terms of CAM use based on trust in conventional medicine and oncological treatments and based on the personal awareness about the disease (Table 5). Considering the 228 patients who did use a CAM: only the 3.9% declared that they had experienced side effects from such therapies [9 patients had diarrhea (G2) during concomitant use of AC and aloe]; 63.1%, on the basis of his/her personal experience do trust these medications; 82% think that the favorable response achieved is due to both the AC and the CAM used; 86.8% admit to having spent between 100 and 300 euros monthly on the chosen CAM. Finally, the multivariate analysis indicated that the only factors which were confirmed to be significantly associated with the use of CAM were a high educational level (Odds Ratio, OR: 1.96 95% Confidence Interval, CI, 1.27-3.05) and receiving treatment in a specialized cancer center rather than in a peripheral hospital/clinic (OR: 2.75 95% CI, 1.53-4.94) (Table 6).

**Table 2:** General feeling about conventional medicine and CAM

General feeling about Conventional medicine and CAM n=468	
***AWARENESS ABOUT DISEASE***	***n (%)***
Absent/poor	47 (10)
Good/excellent	421 (90)
***AWARENESS ABOUT TREATMENTS RECEIVED***	
Absent/poor	28 (6)
Good/excellent	440 (94)
***TRUST IN CONVENTIONAL MEDICINE***	
Yes	441 (94.2)
No	27 (5.8)
***TRUST IN ONCOLOGICAL TREATMENTS***	
Yes	445 (95)
No	23 (5)
***EXPECTATIONS FROM THE ONCOLOGICAL TREATMENT***	
To be treated	236 (50.4)
To be cured	179 (38.2)
To achieve disease stabilization	53 (11.4)
***FINANCIAL SPECULATION AROUND ONCOLOGICAL TREATMENTS***	
Yes	150 (32)
No	318 (68)
***AWARENESS ABOUT CAM***	
Yes	354 (75.6)
No	114 (24.4)
***DIFFERENCE BETWEEN ALTERNATIVE AND COMPLEMENTARY MEDICINE***	
Yes	129 (27.6)
No	339 (72.4)
***SOURCE OF KNOLEDGE ABOUT CAM (n= 354)***	
Media	169 (47.7)
Friends	68 (19.2)
Other	54 (15.3)
Patients	42 (11.9)
Doctors	21 (5.9)
***WHAT CAM ARE (n= 354)***	
Used to reduce the AC toxicities	108 (30.5)
Supplements	50 (14.1)
Anticancer treatments	29 (8.2)
Used to increase the chances of be cured	28 (7.9)
Overall	139 (39.3)

**Table 3:** Personal experience with CAM, distribution of CAM users

Personal experience with CAM n=229	
***REASON FOR USE***	***n (%)***
Anticancer treatment	15 (6.6)
Supportive care	146 (63.8)
Other	68 (29.7)
***TIME OF USE***	
Before the diagnosis of cancer	18 (7.9)
At or after the diagnosis of cancer	211 (92.1)
***DURATION OF USE***	
< 1 year	192 (83.8)
1 - 2 years	29 (12.7)
> 2 years	8 (3.5)
***TYPE***	
Herbal preparations	59 (25.8)
Vitamins	44 (19.2)
Supplements	109 (47.6)
Other CAM	17 (7.4)
***PRESCRIBER***	
Auto-prescription	155 (67.7)
Oncologist	13 (5.7)
Other doctor	13 (5.7)
Friend	42 (18.3)
Relative	6 (2.6)
***AWARENESS ABOUT POTENTIAL SIDE EFFECTS***	
Yes	32 (14)
No	197 (86)
***BENEFIT***	
Yes	79 (34.5)
No	15 (6.6)
Unsure	135 (59)
***SIDE EFFECTS***	
Yes	8 (3.5)
No	221 (96.5)
***DISEASE PROGRESSION DUE TO***	
Disease itself	179 (78.2)
CAM	2 (0.9)
AC	6 (2.6)
Other	42 (18.3)
***DISEASE RESPONSE DUE TO***	
CAM	2 (0.9)
AC	47 (20.5)
Both	180 (78.6)
***ONCOLOGIST INFORMED***	
Yes	196 (85.6)
No	33 (14.4)
***COST***	
< 100 euros	78 (34.1)
100 – 300 euros	144 (62.9)
> 300 euros	7 (3.1)
***PAYED BY***	
Patient	226 (98.6)
National Healthcare Service	3 (1.3)
***CAM EVALUATION***	
Useful as AC treatment	72 (31.4)
Useful as adjuvant treatment	131 (57.2)
Useless	26 (11.4)
***WOULD YOU RECOMMEND CAM***	
Yes	200 (87.3)
NO	29 (12.7)
***TRUST IN CAM***	
Yes	154 (67.2)
No	12 (5.2)
I don't know	63 (27.5)

**Table 4:** CAM use: Association with demographics and disease characteristics

Variable	CAM use		p*
	YES n= 229(%)	NO n= 239(%)	
***SEX***			
male	103(45)	136(56.9)	0.01
female	126(55)	103(43.1)	
***Age***			
< 40	7(3.1)	16(6.7)	< 0.0001
40 – 70	97(42.4)	148(61.9)	
> 70	125(54.6)	75(31.4)	
***EDUCATION***			
Elementary/middle (< 8 ys)	59(25.8)	119(49.8)	< 0.0001
High/degree (< 8 ys)	170(74.2)	120(50.2)	
***ORIGIN***			
Centre/North	173(75.5)	72(30.1)	< 0.0001
South/Islands	56(24.5)	167(69.9)	
***ANXIETY***			
Often	25(10.9)	47(19.7)	< 0.0001
Sometimes	29(12.7)	53(22.2)	
Never	175(76.4)	139(58.2)	
***PAIN***			
Yes	93(40.6)	57(23.8)	< 0.0001
No	136(59.4)	182(76.2)	
***HOSPITAL***			
Cancer centre	202(88.2)	123(51.5)	< 0.0001
Peripheral hospital/clinic	27(11.8)	116(48.5)	
***PRIMARY TUMOUR***			
Breast	24(10.5)	54(22.6)	< 0.0001
Lung	164(71.6)	58(24.3)	
Colorectal	11(4.8)	45(18.8)	
Gastr/Pancr/HCC/Bil tract	5(2.2)	18(7.5)	
Uro-Gynecology	6(2.6)	20(8.4)	
Other	19(8.3)	44(18.4)	
***STAGE AT DIAGNOSIS***			
Early	29 (12.7)	71 (29.7)	<0.0001
Locally advanced	47 (20.5)	67 (28)	
Metastatic	153 (66.8)	101 (42.3)	
***TREATMENT***			
Surgery only	4(1.7)	21(8.8)	< 0.0001
AC only	139(60.7)	99(41.4)	
Surgery +AC	12(5.2)	39(16.3)	
Multimodal	74(32.3)	80(33.5)	
***PS***			
0	1(0.4)	52(21.8)	< 0.0001
1-2	206(90)	140(58.6)	
> 2	22(9.6)	47(19.7)	

**Table 5:** CAM use: Association with awareness about disease and trust in conventional medicine

Variables	CAM use		p*
	YES n= 229(%)	NO n= 239(%)	
***AWARENESS ABOUT DISEASE***			
Absent/poor	26(11.4)	21(8.8)	0.4
Good/excellent	203(88.6)	218(91.2)	
***AWARENESS ABOUT TREATMENTS***			
Absent/poor	8(3.5)	2088.4)	0.026
Good/excellent	221(96.5)	219(91.6)	
***EXPECTATIONS FROM ONCOL. TREATM*.**			
To be treated	161 (70.3)	75 (31.4)	<0.0001
To be cured	43 (18.8)	136 (56.9)	
To achieve disease stabilization	25 (10.9)	28 (11.7)	
***TRUST IN CONVENTIONAL MEDICINE***			
Yes	218(95.2)	223(93.3)	0.4
No	11(4.8)	16(6.7)	
***TRUST IN ONCOLOGICAL TREATMENTS***			
Yes	216 (94.3)	229 (95.8)	0.4
No	13 (5.7)	10 (4.2)	
***FINANCIAL SPEC. AROUND ONC. TREATM*.**			
Yes	44 (19.2)	60 (25.1)	0.009
No	170 (74.2)	148 (61.9)	
Don't know	15 (6.6)	31 (13)	
***SOURCE OF KNOWLEDGE ABOUT CAM***			
Media	133(58.1)	36(28.8)	< 0.0001
Doctors	9(3.9)	12(9.6)	
Patients	18(7.9)	24(19.2)	
Friends	45(19.7)	23(18.4)	
Other	24(10.5)	30(24)	

**Table 6:** Multivariate analysis: High education and receiving treatment in a specialized cancer center are associated with higher risk of CAM use

	CAM users	
	OR (95% CI)*	p-value
***EDUCATION***		
		0.003
Elementary/middle (< 8 ys)	1.0^†^	
High/degree (< 8 ys)	1.96 (1.27-3.05)	
***HOSPITAL***		
		0.001
Peripheral hospital/clinic	1.0^†^	
Cancer centre	2.75 (1.53-4.94)	

*Logistic regression analysis adjusted by terms of gender, age, centre, education when appropriate;^†^ Reference category.

## DISCUSSION

Current international research suggests that 29-91% of cancer patients seek CAM in addition to anticancer therapies [16–23]. Our study demonstrates that CAM use among cancer patients in Italy is fairly widespread, with nearly half of those interviewed (48.9%) reporting an ongoing or recent use of CAM. Patients suffering from cancer are more likely to use CAM at some point in their disease history however, many available publications show that unconventional medicine is routinely used by many other categories of patients: the average lifetime prevalence for UK patients reported by Posadzky P et al. [7] is 51.8%; another work published in 2015 [16] analyzing the data from 263 questionnaires completed by New Mexican patients attending outpatient clinics for different diseases (heart failure, HIV, hepatitis, etc.), found a 56% incidence of CAM use with a significantly higher rate among females with a high educational level, who declared that they use CAM for general wellness and disease prevention. The study by Zhang Y et al. evaluating a large population of adults in the US (34.525) also concluded that CAM use was more popular among females with a higher educational level; in that work, the main problems leading to CAM were back pain, arthralgia, and migraine [24]. Looking at the literature about the oncological population, our findings suggest a higher rate of use compared to previous European studies, which appears close to the prevalence data observed in several US studies. Maggiore RJ et al. presented the results of a secondary cross sectional analysis that examined medication use in 545 ambulatory senior adults in the context of a multicenter, longitudinal study evaluating the utility of a comprehensive geriatric assessment in predicting AC toxicity among a cohort of older adults with cancer [12]. As part of the study, the patients were asked to list all their medications, including herbal preparations, vitamins and supplements; the mean age of the cohort was 73 years with 52% being women; the reported CAM prevalence was 17%, with a mean of two herbal medications (range, 1–10), CAM use was shown to be more common among those who had an earlier cancer stage (i.e., those receiving adjuvant, potentially curative treatment, OR 2.05; 95% CI, 1.21-3.49) and higher functional status (less impairment with instrumental activities of daily living, OR 1.39; 95% CI, 1.12-1.73), no difference was noticed between males and females. In an abstract presented by Lichtman SM et al. [13] at ASCO 2009, the prevalence of herbal/vitamin use was 46% in a cohort of 154 cancer patients aged ≥ 65 years of age, no information were given about potential association between CAM use and sex, educational level and/or cancer stage. In a retrospective study by Sokol KC et al. [14], considering 100 ambulatory senior adults with cancer (mean age 78 years, range: 70–90), the reported rate of CAM use was 46%, slightly lower compared to to our finding. Wyatt GK et al. [15] reported the data of a survey carried out among 699 cancer patients who were receiving AC, 33% declared that they use complementary therapies. The characteristics associated with CAM use were: being female, having breast cancer and high level of education; the most frequent practices were exercise, herbal therapy, and spiritual healing; there were no differences with respect to reported depressive symptomatology or spirituality. Nightingale G et al. [25] explored the prevalence of CAM and factors influencing CAM use in a secondary analysis of 248 senior adults with cancer (mean age 79.9) who received an initial comprehensive geriatric oncology assessment; 234 subjects were included in the final analysis. In that study, the prevalence of CAM use was much lower compared to our finding, 26.5%; polypharmacy, vision impairment and urologic comorbidities were found associated with CAM use. Such a difference in terms of prevalence of CAM use is in part justified by the direct involvement of a clinical pharmacist who went through the complete list of medications of each patient and actively helped to define what CAM was; also, vitamins were excluded. In regards to patient demographics, a higher rate of CAM use was observed in women (19% vs. 8%) though the population was not well balanced (64% females, 36% males); the educational level was not mentioned. An interesting study involving 294 prostate cancer patients reported a 25% rate of CAM use, mainly vitamins, low-fat diets, lycopene and green tea. The multivariate analyses revealed no differences in mental health scores between users and non-users; users were younger (OR 0.93, 95% CI 0.89–0.97) and more likely to be receiving ‘active surveillance’ rather than treatment (OR 5.23, 95% CI 1.78–15.41). An important finding was that nearly half of the users, 43%, had not informed any doctor about the product taken [25]. The European survey published by Molassiotis A et al. [26] included 956 patients from fourteen countries. Approximately one third of the interviewed, 35.9%, said that they were CAM users, with a higher rate in Italy (the Italian data were only on 52 patients treated with palliative care); the three variables strongly associated with CAM use were female gender, younger age and a high educational level. An interesting survey by Patterson RE et al. [27] involving 356 patients with colon, breast, or prostate cancer identified from the Cancer Surveillance System of western Washington showed a prevalence of CAM use up to 70.2% with females five times more likely to see an alternative provider and about twice as likely to use mental therapies or supplements (p < 0.05 for all). Also younger age and higher education were associated with the use of all types of CAM; general health and well-being were the main reasons for CAM consumption. In the work by Richardson et al. [28], 83.3% of the responders had used at least one CAM approach, use was greatest for spiritual practices (80.5%), vitamins and herbs (62.6%), and movement and physical therapies (59.2%) and was predicted (P <.001) by sex (female), younger age, indigent pay status, and surgery. Oneschuk et al. [29] (Canada) published their findings from 143 advanced cancer patients attending an outpatient pain and symptom clinic at a regional cancer center in which they found a 37% rate of CAM use: 39.6% herbs, 32.5% vitamins, 6.6% minerals, 10.7% other medications including shark cartilage, and 10.7% could not be identified. Both the anticancer effect and the promotion of well-being were prominent among the stated reasons for using these medications. Our study has corroborated some findings already observed in previous papers. Firstly, the higher prevalence of CAM use among females and, in general, in the subset of patients with a high educational level. Probably a high education level allows easier access to the media, internet and information about medicine. An aspect that has emerged in our dataset is the higher rate of CAM use in patients receiving their treatment in highly-specialized cancer centers rather than in peripheral hospitals/clinics. In the univariate analysis we also found that patients with anxiety disorders were more likely to use CAM though this association was not confirmed by the multivariate analysis; however, this lack of statistical significance may be due to the low number of patients suffering from anxiety present in our study (only 15.4%, 72/368). Our data are also consistent with the available literature regarding the absence of a relationship between CAM use and lack of trust in conventional medicine, other authors, in fact, have already observed that CAM are often used by patients who are fully satisfied with conventional health care and look for adjuvants rather than for substitutes to traditional practices [29, 30]. As observed in previous studies [31–34] supplements and herbal medications are the main CAM used by cancer patients and they are more likely to be used as adjuvants, to reduce AC side effects, rather than as direct anticancer treatments. In contrast with prior observations, 85% of our interviewed subjects said that they had informed their oncologist about their CAM use, though such data should be carefully interpreted due to the administration of our questionnaire by physicians. The costs sustained to buy CAM were between 100 and 300 euros per patient, per month for most patients (62.9%) and were paid for by the patients themselves in 98.6% of cases. Such finding is consistent with the estimated cost per year of supplements in Italy which accounts up to 2.5 billion of euros [35]. The most CAM used was aloe (75% of patients). In 67.7% of the cases, the CAM prescription was a self-prescription and only in 11.4% of the cases had a doctor prescribed the CAM. This aspect is very dangerous considering the risk of toxicities and potential interaction between CAM and standard/conventional medicine; regarding this aspect, 197 patients (86%) did not know about the risk of potential side effects. Finally, the high rate of satisfaction from CAM and the fact that approximately all the users (87%) recommended their use, indeed, deserves attention; maybe healthcare professionals in general and oncologists in particular should pay more attention to this aspect of the anamnesis at the time of the visit and check at each control, especially for patients on AC, if new conventional or unconventional drugs have been added to the patient's personal therapy. The routine anamnesis collection should be completed by explicit questions about the use of herbals, supplements, and any other preparations. In fact some CAM products can interfere with some AC through effects on the metabolic pathway and oncologists must be careful to avoid drug-herbs interaction when they plan a treatment with herbal medications as supplement to AC (Table 7).

**Table 7:** The most common botanicals used in cancer patients and their possible interactions with drugs [39–43]

Agents	Effect on metabolic pathway	Interaction with anticancer drugs
Ananas (Bromeline)	CYP2C9 inhibition	Risk of over dosage with paclitaxel
Curcuma	CYP1A2, CYP2B6, CYP2C9, CYP2D6 weak inhibition	Risk of over Dosage with Bendamustine, Risk of inefficacy of pro-drugs (Ciclophosphamide, Tamoxifen etc)
Cannabinoides	CYP2C9 induction	Risk of over dosage of prodrugs (Ciclophosphamide, Tamoxifen etc)
Echinacea	CYP3A4 induction	Improved pharmacokinetic (weak) of Ciclophosphamide dasatinib, docetaxel, erlotinib, imatinib, sorafenib, vinca alkaloides
ESSIAC*	CYP3A4 inhibition	Risk of over Dosage with bortezomib, dasatinib, docetaxel, erlotinib, imatinib, sorafenib, vinca alkaloides
Green Tea	CYP3A4 inhibition	As for Essiac
Gingko Biloba	CYP3A4 CYP2C19, inhibition	As for Essiac
Grape Fruit	CYP3A4 inhibition	As for Essiac
Licorice	CYP2B6, CYP3A4 weak inhibition	As for Essiac (weak)
milk thistle	CYP2C8, CYP2C9 weak inhibition	Risk of over Dosage with ciclophosphamide, paclitaxel
St. John's worth (Hypericum)	CYP3A4 induction	Improved pharmacokinetic of Ciclophosphamide dasatinib, docetaxel, erlotinib, imatinib, sorafenib, vinca alkaloides

**Additional Source:** http://reference.medscape.com/drug-interactionchecker.

**Abbreviations:** Cytochrome P450: CYP.

*Essiac: herbal mixture patented as anticancer therapy in the 1920 by Rene Caisse in Canada.

The use of CAM in oncologic patients is a growing issue that requires more attention by the scientific world and physicians. To know the causes that induce oncologic patients to inappropriately use CAM is of fundamental importance. To retrieve the distortion of the information sources and to improve the communication modalities are crucial steps to preserve the health of patients, trough the strengthening of the therapeutics alliance and of the adhesion to treatment with reliable efficacy. We firmly believe that to promote knowledge about CAM between patients and physicians with the aim of alerting them to potential toxicities is very important. The need to increase the evidence base of CAM therapies, using methodologies that are appropriate and sensitive to CAM cannot be overemphasized. This is also important from an economic point of view, as its use is a multibillion Euro business, and, as shown in this study, some patients pay large sums out of their pockets to receive such interventions. In the US, where such data are available, the use of CAM is conservatively estimated to cost patients US$ 27 billion (for the year 1997) [36]. In the EU it is the second biggest growth industry [37]. Finally, appropriate legislation and regulation of CAM therapies in the EU is also necessary.

## MATERIALS AND METHODS

### Patients setting

A cross-sectional descriptive survey design was used to collect data trough a questionnaire about CAM therapies. Nine hospitals with oncology and/or radiotherapy divisions were approached for possible collaboration in this study. Of the nine hospitals, five agreed to participate. A key person from each hospital was selected, based on interest in and/or experience with CAM. Data collected from each hospital were returned to one of the investigators and then the data were coded for analysis. This survey was approved by the ethics committees and carried out between September 2014 and May 2015. Both metastatic and non-metastatic cancer patients were included from cancer hospitals, oncology units of general hospitals, day units, and radiotherapy units. All participating patients received information about the study. Patients were considered eligible if they met the following inclusion criteria, they were Italian-speaking, adult patients of either gender with a diagnosis of cancer; aware of their diagnosis; were receiving an anticancer treatment [antiblastic chemotherapy (AC), endocrine therapy, radiotherapy and surgery] at the time of the survey or had received an anticancer treatment no more than three years before participating in the survey; able to understand the questions; free from any condition that would make completing the questionnaire inappropriate or overburdening for the patients; and they were willing to participate in the study.

### Procedures

The questionnaire was administered to the patients after they received information about the study, agreed to participate, and signed the consent form. Patients completed the questionnaire with the support of a physician. Participation was voluntary and did not interfere with medical treatment.

### The questionnaire

The questionnaire used was based on the one developed by Molassiotis A et al. [26]. However, the questionnaire was modified for the purpose of the present study and some new items were added (for example: awareness about the disease and treatment received, trust in conventional medicine and oncological treatments, expectations from oncological treatments, awareness about CAM, knowledge about the differences between alternative and complementary medicine, source of knowledge about CAM and role of CAM in oncological treatment), and some others were modified to reflect Italian culture (for example: geographic area of origin) there were 42 items in total. These included demographic data (age, gender, occupation, and education), clinical data (site of primary cancer, standard treatments received previously and current standard treatment) and questions about CAM use, in particular: knowledge about what CAM is and whether it has ever been used. The questionnaire about CAM was completed only by the patients who declare that they have had, or had at the time of the survey, a personal experience with CAM and included eighteen questions about different aspects of such therapies concerning, e.g. efficacy, cost, side effects, satisfaction rating. On the basis of the available literature we decided to exclude from the analysis medical therapies routinely prescribed, such as iron, vitamin D, and calcium supplements, which are often used as support therapy.

### Data analysis

In the descriptive analysis, the median, range, and relative frequencies were used. Analyses of frequencies and cross tables with χ^2^ tests were done using IBM SPSS Statistics 20 [38]. In this study, we focused on those questions that were the same or similar in both group in order to compare patients and professionals. Only completely filled-out questionnaires were analyzed.
